# Promising Approaches for Engaging Youth and Young Adults Living with HIV in HIV Primary Care Using Social Media and Mobile Technology Interventions: Protocol for the SPNS Social Media Initiative

**DOI:** 10.2196/10681

**Published:** 2019-01-31

**Authors:** Melissa Medich, Dallas T Swendeman, W Scott Comulada, Uyen H Kao, Janet J Myers, Ronald A Brooks, Special Projects of National Significance Social Media Initiative Study Group

**Affiliations:** 1 Department of Family Medicine University of California, Los Angeles Los Angeles, CA United States; 2 Department of Psychiatry and Biobehavioral Sciences University of California, Los Angeles Los Angeles, CA United States; 3 Department of Medicine University of California San Francisco San Francisco, CA United States

**Keywords:** HIV, health outcomes, mobile technology, social media, youth, young adult, mobile phone

## Abstract

**Background:**

In the United States, disparities in the rates of HIV care among youth and young adults result from the intersections of factors that include stigma, substance use, homelessness or marginal housing, institutional neglect, and mental health issues. Novel interventions are needed that are geared to youth and young adults.

**Objective:**

In this paper, we aim to describe the interventions used by participating sites for Using Social Media initiative, the process for classifying the intervention components, and the methods for conducting a comprehensive evaluation of the interventions.

**Methods:**

In 2015, the Health Resources and Services Administration (HRSA) HIV/AIDS Bureau, Special Projects of National Significance (SPNS) funded the Evaluation and Technical Assistance Center (ETAC) at the University of California, Los Angeles and 10 demonstration projects at sites across the United States that incorporated innovative approaches using a variety of social media and mobile technology strategies designed specifically for youth and young adults living with HIV. The ETAC developed a typology, or a classification system, that systematically summarizes the principal components of the interventions into broader groups and developed a multisite, mixed-methods approach to evaluate them based on the Department of Health and Human Services HIV health outcomes along the HIV care continuum. The mixed-methods approach is key to remove potential biases in assessing the effectiveness of demonstration projects.

**Results:**

This SPNS project was funded in September 2015, and enrollment was completed on May 31, 2018. A total of 984 participants have been enrolled in the multisite evaluation. Data collection will continue until August 2019. However, data analysis is currently underway, and the first results are expected to be submitted for publication in 2019.

**Conclusions:**

This HRSA-funded initiative seeks to increase engagement in HIV medical care, improve health outcomes for people living with HIV, and reduce HIV-related health disparities and health inequities that affect HIV-positive youth and young adults.

**International Registered Report Identifier (IRRID):**

DERR1-10.2196/10681

## Introduction

### Background

In the United States, HIV-positive youth and young adults have disproportionately lower rates of HIV care engagement, retention, medication adherence, and viral suppression compared with older HIV-positive populations. Zanoni and Mayer [1] estimate that only 25% of HIV-positive youth and young adults in the United States are linked to care, 11% retained in care, and 6% virally suppressed. Data on HIV clinical outcomes among youth are limited, but their retention-in-care rates suggest that they are less likely to be meaningfully engaged in care and to achieve viral suppression [2-4]. Although current antiretroviral therapy regimes are less toxic and simpler, factors that are more likely to be present in younger populations are also associated with suboptimal adherence [2,5,6]. HIV-positive youth and young adults face HIV-related health disparities resulting from the intersections of multiple and concurrent stigmas (eg, homophobia, race or ethnicity, and HIV), substance use, homelessness or marginal housing, institutional neglect, mental health issues, and other challenges [2,5,6].

The largest percentage of HIV-positive youth and young adults are men who have sex with men (MSM). Among Ryan White HIV/AIDS Program (RWHAP) clients aged 13-30, male-to-male sexual contact was the transmission category for 60% of HIV infections, with African Americans (54%) and Latinos (22%) being the largest racial or ethnic groups affected [7]. The RWHAP is a federally funded program, authorized by title XXVI of the Public Health Service Act, which provides a comprehensive system of care–primary medical care and support services–for people living with HIV who are uninsured or underinsured. Challenges to engagement in HIV care and viral suppression for young MSM include substance use disorders, mental health issues, stigma, discrimination, and marginalization [8]. Stigma resulting from an HIV-diagnosis and fear of familial, peer, and community rejection profoundly impact youth and young adults [9] and is associated with higher rates of depression, anxiety, and social isolation [10-12]. Aspects of the health care environment exacerbate care engagement challenges because medical providers often reproduce and communicate larger social homophobia and HIV-related stigma [13,14]. Furthermore, there are structural barriers that limit access to HIV care, such as limited health care insurance and lack of transportation, especially among those with a low income and racial or ethnic minorities [14-16].

Most interventions that address barriers to HIV care have been developed for adults [1] and have not been tailored to youth struggling with a variety of unique issues including identity formation, economic hardship, and unstable housing among other daily survival issues [1,7]. A recent randomized control trial attests the importance of tailoring medication adherence interventions to an increasingly young HIV-infected population [17]. However, one promising new strategy with the potential to help young people overcome these challenges is the use of mobile technology. The type of technology use among youth is constantly increasing, and new forms of communication technology and Web-based social networking offer opportunities to reach and engage young people for health promotion [18-20].

### Mobile Technology and Social Media

Significant gains can be made to improve the health outcomes of HIV-infected youth and young adults using mobile technology and social media for engagement and retention in HIV medical care. Media and technology that facilitate social interaction (ie, social media) are preferred among young adults, who spend more time with social media and mobile technology than any other activity [21,22]. The science and practice of leveraging social media and mobile apps to support youth in accessing care hold great promise for better patient outcomes. A growing body of evidence suggests that mobile app interventions and social media can help in achieving HIV care program priorities, including linkage to care, engagement and retention in care, and adherence to HIV medications [23-26]. Significant advantages to using mobile technologies and social media apps for engagement and retention in HIV primary care include convenience to the user, reaching larger numbers of people, consistency in delivery, real-time exchange, and potential privacy protections [17,27]. Smartphones have revolutionized the mobile communications markets, and mobile phone health interventions are increasingly being used for the care and prevention of HIV and other sexually transmitted diseases [28-31]. Over 95% of all Americans own a cell phone [32], and over 3-quarters (77%) own a smartphone; the majority (92%) of smartphone owners are between the ages of 18 and 29 years [32].

### Overview of the Special Projects of National Significance Social Media Initiative

To harness and test the potential of social media in the interest of better HIV care engagement for youth, the Health Resources and Services Administration (HRSA) HIV/AIDS Bureau, Special Projects of National Significance (SPNS) program launched the Using Social Media to Improve Engagement, Retention, and Health Outcomes along the HIV Care Continuum initiative (SMI) in 2015. The 4-year initiative includes ten demonstration projects in HIV care sites (n=5), health departments (n=2), and community-based organizations (n=3) located throughout the United States. An Evaluation and Technical Assistance Center (ETAC) was awarded to the Department of Family Medicine at the University of California, Los Angeles (UCLA) to provide technical assistance to the demonstration projects and to develop and implement a rigorous multisite evaluation. Demonstration projects developed mobile technology and social media interventions for linking, retaining, and supporting HIV-positive, underserved, underinsured, hard-to-reach youth (ages 13-24 years) and young adults (ages 25-34 years) in HIV primary care and supportive services. The overarching goal of this initiative is to create a system change—improvements in policies and procedures using social media and mobile technologies—that results in improved HIV health outcomes for HIV-positive youth and young adults.

In this paper, we describe the interventions used by participating sites for this initiative, the process for classifying the intervention components, and the methods for conducting a comprehensive evaluation of the interventions.

## Methods

### Demonstration Sites

Through a competitive proposal process, HRSA selected demonstration sites using innovative mobile technology and social media strategies deployed via the internet or mobile apps designed to improve engagement and retention in care, medication adherence, and to help achieve viral load suppression among youth and young adults living with HIV (see Multimedia Appendix 1 for a more detailed description of the interventions). The ten demonstration sites are located in Los Angeles, California; San Francisco, California; Chicago, Illinois; St Louis, Missouri; Winston-Salem, North Carolina; New York, New York; Cleveland, Ohio; Hershey, Pennsylvania; Philadelphia, Pennsylvania; and Corpus Christi, Texas. Each demonstration site used its own outreach, linkage, and retention strategies tailored to their local target populations. They all used youth advisory boards either to modify and tailor existing intervention approaches or to develop new intervention approaches for the populations they are serving.

### Target Population

The initiative focuses on youth and young adults. HRSA defined youth as persons between the ages of 13 and 24 years and young adults as persons between the ages of 25 and 34 years. The sites focused on the age ranges of their target population based on the groups most affected by HIV in their local communities. The SMI includes all genders, races or ethnicities, and sexual orientations. Nonetheless, some interventions focus on specific populations, such as transgender women, MSM, or MSM within specific racial or ethnic groups such as African American or Latino. Demonstration sites classified and described the respective target population for their intervention by setting, age, gender, race or ethnicity, and sexual orientation.

### Youth Involvement

An important component of this initiative is the involvement of youth advisory groups providing input in the design of intervention and outreach strategies, typically via focus group discussions. Engaging the target population to guide intervention design is important in developing an intervention that resonates with them and ensures cultural and linguistic appropriateness crucial to the development of messaging in social media-based components [33]. Across the demonstration sites, youth and young adults have been engaged in multiple ways such as guiding the process and design of messages that market the interventions to potential users and providing feedback on the content of intervention messaging and informing app feature priorities and functions. Demonstration site staff typically recruited youth to attend regular meetings to ensure young people had consistent opportunities to provide input in developing components of the intervention and to gather feedback about the effectiveness of implemented strategies. Youth advisory groups give voice to young adults’ own lived experiences from different regions in the United States, serving as an important step forward in understanding the connection between social media and technology use and young adult health behaviors [33]. Thus, the involvement of youth in the project design is critical for sustainability and meaningful, long-term impact.

### Social Media Initiative Interventions Typology

The UCLA ETAC reviewed each of the funded proposals and their intervention descriptions to establish a classification system that systematically summarizes the main components of the interventions into a typology. We chose this approach because the use of typologies has proved more useful than hierarchies of evidence (systematic reviews, meta-analyses, randomized controlled trials, cohort studies, etc) in conceptualizing the strengths and weaknesses of different methodological approaches [34]. In other words, hierarchies of evidence misrepresent the interplay between the question being asked and the type of approach most suited to answering it. Typologies systematically indicate the relative contributions that different kinds of methods can make to different kinds of research or, in this case, evaluation questions [35]. The typology developed includes a description of the target population, inclusion criteria, intervention components and functions, and how these correspond to the HIV health outcomes along the HIV care continuum. The typology provided a framework to develop the multisite evaluation of these interventions, discussed below.

Table 1 includes information on the respective target populations by setting, age, gender, race or ethnicity, and sexual orientation for each demonstration site. Most sites target similar populations, with few exceptions. There is one demonstration site that is a community research site, two are Departments of Health, one is a hospital system, and the rest are clinics in either a community or a university setting. There are 6 sites that target participants under the age of 18, while the others focused on ages 18-34 years. There is one site where the intervention is designed specifically for transgender women and another specifically for men, while half are designed for all genders and sexual orientations.

#### Inclusion Criteria for Enrollment in the Multisite Evaluation

HIV medical eligibility criteria for enrollment in the multisite evaluation is based on the US Department of Health and Human Services (HHS) common core indicators for monitoring HHS-funded HIV care services [36] and include (1) being newly diagnosed, which is defined as testing HIV positive for the first time within the last 12 months prior to enrollment; (2) not being linked to HIV medical care, including participants who are aware of their HIV infection status but have never engaged in care (never having an HIV medical visit after being diagnosed with HIV); (3) being out of care or not fully retained in care, which includes participants diagnosed with HIV more than 12 months prior to enrollment who had a gap in their HIV care that was >6 months, within the last 24 months; or (4) not being virally suppressed, defined as having a viral load of ≥200 copies/mL at their last lab test.

**Table 1:** Target populations for the Using Social Media to Improve Engagement, Retention, and Health Outcomes along the HIV Care Continuum initiative (SMI).

Demonstration site	Setting	Age (years)	Gender	Race or ethnicity	Sexual orientation
Coastal Bend Wellness, Corpus Christi, Texas	Community clinic	13-34	Male and female	All (focus on African American and Latino)	All
Friends Research Institute, Los Angeles, California	Community research site	18-34	Transwomen	All	All
Howard Brown Health Center, Chicago, Illinois	Community clinic	13-34	Male and transwomen	All	MSM^a^ and heterosexual
MetroHealth System, Cleveland, Ohio	Hospital system	13-34	All	All	All
New York State Department of AIDS, New York, New York	Health department	18-34	All	All	All
Pennsylvania State University Medical Center, Hershey, Pennsylvania	Community and university clinic	13-34	All	All	All (primarily MSM^a^)
Philadelphia FIGHT and Children’s Hospital of Philadelphia, Philadelphia, Pennsylvania	Community clinic	14-29	All	All	All
San Francisco Department of Public Health, San Francisco, California	Health department	18-34	All	All	All (primarily MSM^a^)
Wake Forest University, Winston-Salem, North Carolina	University clinic	13-34	Male	All	MSM^a^
Washington University St Louis, St Louis, Missouri	University clinic	18-29	Male and female	All (primarily African American)	MSM^a^ and heterosexual

^a^MSM: men who have sex with men.

Additional eligibility criteria included (1) being between the ages of 13 and 34 years; (2) meeting at least one of the above medical criteria determined from tests or medical records; (3) providing informed consent (if 18 years or older) or providing informed assent (if 13-17 years) and, if required by state laws and regulations, obtaining consent from a parent or legal guardian; and (4) meeting any demonstration site-specific criteria (eg, smartphone ownership or being a patient at the site’s clinic) as necessary.

#### Technology Platforms

While each demonstration site’s intervention is unique, there are general commonalities listed in the typology framework (Table 2). For example, all ten demonstration sites include a text messaging service component, three of which use text messaging services exclusively. Text messaging is done through short message service (SMS) or private messaging apps such as WhatsApp [37] or Kik [38], while private messaging also functions in mobile Web apps or social media apps and sites. There are six sites that developed new mobile apps specifically for their intervention, while one site has adapted an existing mobile app. The different technology platforms used in each intervention are described in Table 2.

For outreach and recruitment, almost all (n=9) of the sites use social networking sites or apps and other social media platforms (Table 2). Of these, seven have corresponding websites, with three using apps optimized for mobile devices to support the promotion of their interventions. There is one site that uses YouTube, Twitter, and Instagram as platforms for their graphic serial.

#### Functions of the Interventions

The interventions offer a range of functions, as represented in Table 3. Most interventions have seven to nine functions (an average of seven functions). There is one intervention that has only one function, namely, automated information delivered through SMS, and one other intervention contains all ten functions. The most common components of the interventions are communication, information, social support or networking, and reminders for HIV medical care appointments, HIV medication, and non-HIV care-related issues. The least common components are the skills building and gaming components. In general, youth advisory groups across the ten demonstration sites communicated in focus groups during formative research that medical appointment reminders and support for medication adherence were most important, followed by receiving lab results for HIV viral load. As a result, most interventions focus on helping participants to develop good habits relating to retention in medical care, medication adherence, and monitoring viral load. Developing skills and gaming are functions of interventions that target younger youth (13-24 years of age). The idea of gaming includes interactive games, quizzes, and puzzles and a points system for the use of the mobile app in achieving appointment, adherence, and viral suppression milestones. There is one site where the mobile app offers immediate feedback and incentives while using avatars to be more attractive to their target population.

**Table 2:** Technology platforms for the Using Social Media to Improve Engagement, Retention, and Health Outcomes along the HIV Care Continuum initiative (SMI).

Demonstration site	Text messaging	Mobile apps	Social networking sites or apps	Social Media^a^	Website
Coastal Bend Wellness, Corpus Christi, Texas	All types	—^b^	✓	✓	✓^c^
Friends Research Institute, Los Angeles, California	Automated, unidirectional	—	✓	✓	—
Howard Brown Health Center, Chicago, Illinois	Automated	✓ (adapted)^d^	—	—	✓
MetroHealth System, Cleveland, Ohio	Automated	✓ (new)	—	✓	✓
New York State Department of AIDS, New York, New York	All types	✓ (new)	✓	✓	✓^c^
Pennsylvania State University Medical Center, Hershey, Pennsylvania	All types	✓ (new)	✓	✓	✓^c^
Philadelphia FIGHT and Children’s Hospital of Philadelphia, Philadelphia, Pennsylvania	All types	✓ (new)	✓	✓	✓
San Francisco Department of Public Health, San Francisco, California	Live, bidirectional	✓ (new)	✓	✓	✓
Wake Forest University, Winston-Salem, North Carolina	Live, bidirectional	—	✓	✓	—
Washington University St Louis, St Louis, Missouri	All types	✓ (new)	—	✓	—

^a^Facebook, Instagram, Twitter, Snapchat, and YouTube.

^d^Not applicable.

^c^Mobile optimized.

^d^Adapted apps signify that institutions have existing mobile apps that they have adapted for this intervention and target population.

**Table 3:** Intervention functions of the Using Social Media to Improve Engagement, Retention, and Health Outcomes along the HIV Care Continuum initiative (SMI).

Function	Definition	Interventions (N=10)
Communication	Interactive communication between participants and service providers.	9
Education	Interactive teaching of information or content.	6
Gaming	Rewards, incentives, or a points system embedded in the social media/mobile digital tool that may or may not include competition between peers.	2
Information	One-way or “push” of content to inform participants (eg, tips, referral resources)	9
Skills building	Social media tools specifically designed to build skills through demonstration and practice.	3
Social support or social networking	Provides participants with opportunities to receive social support from peers, family, service providers, or others.	9
General reminder	Reminders other than for HIV care appointments or HIV adherence.	9
Medical appointment reminder	Appointment reminders for HIV medical care, delivered via the social media intervention tool (can be automated).	9
Medication adherence reminder	Antiretroviral medication reminder that can by automated, live, or both.	8
Monitoring or tracking reminder	Participants record or report information via the social media tools (ie self-monitoring, logging, self-tracking)	7

**Table 4:** Data collection tools for the Using Social Media to Improve Engagement, Retention, and Health Outcomes along the HIV Care Continuum initiative (SMI).

Methods	Time frame
Audio Computer-Assisted Self-interview Surveys	Baseline and 6, 12, and 18 months
Cost assessments	Annually
Intervention exposure	Monthly or every encounter
Back-end data	Weekly
Medical chart data	Every 6 months

### Comprehensive Evaluation Strategy

In order to determine the relative effectiveness of the interventions taking part in this initiative, the ETAC is conducting a rigorous, multisite evaluation of the demonstration sites’ interventions. The evaluation plan assesses the outcomes, processes, and cost of using social media- and technology-based interventions to ensure that they have maximum impact on engagement, retention, adherence, and health outcomes of HIV-infected youth and young adults. The design of the quantitative multisite evaluation is informed by the components of each site’s interventions and the type of data the components capture (eg, intervention exposure such as back-end data or person-to-person contact by intervention staff) as well as engagement and outcomes of care measures approved by HHS for funded HIV care services [39]. The demonstration sites recruited a convenience sample of 984 participants across a 20 month period from October 2016 through May 2018 (see Inclusion Criteria above). Intervention participant data is being collected using audio computer-assisted self-interview (ACASI) survey software to increase privacy and confidentiality in the data collection process. Table 4 provides a list of the data collection tools being used in the multisite evaluation. All data are submitted to the ETAC through a Web-based secure portal at UCLA for data management and analysis.

#### Audio Computer-Assisted Self-Interview Surveys

ACASI surveys are conducted at baseline enrollment and repeated at 6-, 12-, and 18-month intervals. The five primary domains of the ACASI surveys are: (1) sociodemographic characteristics (eg, age, education, housing stability, and incarceration); (2) biomedical health, linkage, engagement, and retention in care; (3) intervention exposure; (4) barriers to care; and (5) media technology usage and attitudes. In addition, the surveys collect information on the popularity, adoption, and usability of social media-based interventions among participants. Surveys also gather information on the broader barriers and facilitators of engagement and retention in medical care.

#### Cost Assessments

Cost assessments are being conducted annually to determine the cost of implementing each intervention. Sites use standard microcosting techniques (incremental time required for each intervention) combined with direct costs to obtain an estimate of total incremental recurring costs. Development and ongoing maintenance costs of the studies are collected through a cost assessment tool that captures personnel information, recurring goods and services, capital equipment, and facility costs. The costs of developing new mobile apps are captured in the recurring costs reported by the five sites developing them. Cost assessments also indicate successful strategies for labor and programmatic costs for each intervention in this SPNS initiative to inform future replication.

#### Intervention Exposure: Back-End Data and Person-to-Person

Intervention exposure collected weekly helps identify which components of the interventions contribute to desired outcomes. There are two forms of exposure data being captured in the SMI: person-to-person and back-end data. Person-to-person exposure is defined as any type of contact between participants and intervention staff in person, by phone, by text, or by other mobile messaging services. Back-end data include participants’ activities on mobile apps, private Facebook pages, or other social media platforms used in this intervention. Back-end data will be used to measure intervention exposure in the multisite evaluation. Some sites have used real-time measures of back-end data to adjust their intervention. For example, one demonstration intervention removed the gamification component of their mobile app due to lack of use.

#### Medical Data

Data about participants’ HIV health outcomes (medical data) are collected every 6 months to assess changes in health outcomes over time. Sites use either the administrative data associated with the receipt of RWHAP funds or abstracted data collected from medical records by hand. Participants’ identification is coded to protect their identities before sites submit data to the ETAC. Information from medical data includes: core service visits for HIV care, substance abuse, mental health, CD4 cell count testing, viral load testing, the first date of antiretroviral prescription, and any breaks in the use of highly active antiretroviral therapy.

#### Qualitative Methodology

Qualitative analysis will document the effective implementation of these interventions. Data is being collected by ETAC investigators during years three and four of the initiative through key informant interviews with participants and providers and tracking reports and forms kept by demonstration sites. Qualitative research methodologies are valuable for understanding factors that facilitate or inhibit the implementation and the effectiveness of the intervention, thus providing context and informing quantitative HIV health outcome data *.* In addition, qualitative methods afford a better understanding of participants’ experiences with social media and mobile technology to link, engage, and keep them in HIV medical care. It also provides a means to capture any unanticipated themes that may emerge from the data regarding intervention implementation and acceptability.

#### Multisite Evaluation

The multisite evaluation is assessing engagement in care, health outcomes associated with participation in the social media and mobile technology-based interventions, and individual-level factors that influence the effectiveness of these interventions.

The quantitative evaluation primarily looks at associations between intervention type and exposure and changes in HIV care continuum outcomes and related health outcomes over time. Statistically significant changes in outcomes over time will be indicative of a possible intervention effect; we are cautious against using more causal language in the absence of a control group. We will conduct subgroup analyses for data from each of the ten demonstration sites as well as in aggregate to evaluate differential intervention effects across sites. Random effects will be included for each study participant to account for correlations between outcome observations on the same study participant and properly adjust SEs that will be estimated by the regression models. Most of the analyses will be conducted on nonnormally distributed outcomes and will use random effects generalized linear models with appropriate outcome link functions.

The qualitative evaluation will document the barriers and facilitators to the effective implementation of interventions. Qualitative data sources include individual, semistructured interviews with participants or clients and key informants (site staff implementing the social media interventions), review of secondary sources of information (eg, demonstration site grant proposals, notes from ETAC site liaisons, and ETAC site visit reports) and site presentations at grantee meetings, and observations of project operations at intervention sites. Interview transcripts will be iteratively coded, sorted, and analyzed using a thematic analysis process [40]. Themes will be selected based on their prevalence across the dataset and importance in assessing barriers and facilitators to implementation and acceptability among participants.

This mixed-methods approach will be important in removing potential bias in establishing the effectiveness of demonstration projects. The findings from the evaluation will provide insight for the future use of social media and mobile technology to improve health outcomes for HIV-positive youth and young adults. The results will include best practices from the demonstration sites, lessons learned, and implications for system change or system integration of social media and mobile technology.

#### Privacy, Confidentiality, and Security

Privacy, confidentiality, and security are paramount in designing an intervention that uses social media or mobile technology for engaging and retaining HIV-infected persons in medical care. In addition to firewalls and information technology security procedures required by the Health Insurance Portability and Accountability Act (HIPAA) regulations, unintentional disclosures can result in stigma, discrimination, and prejudice, particularly for HIV-positive patients. Participants in the SMI are trained in device security, such as using passwords to access phones, added layers of password protection to access specific apps, how to clear text message logs, etc, by SPNS staff at the demonstration sites. Awareness of current privacy concerns associated with each technology has helped alleviate participant concerns. HIPAA requirements must be adhered to when using mobile technologies and social media connected to patients’ personal health information.

## Results

This SPNS project was funded in September 2015, and enrollment was completed on May 31, 2018. A total of 984 participants have been enrolled in the multisite evaluation. Data collection will continue until August 2019. However, data analysis is currently underway, and the first results are expected to be submitted for publication in 2019.

## Discussion

The Social Media Initiative is an HRSA SPNS initiative that emphasizes the primary goals for HIV prevention and care outlined in the US National HIV/AIDS Strategy: to reduce new infections, increase access to care, improve health outcomes for people living with HIV, and reduce HIV-related health disparities and health inequities that HIV-positive youth and young adults face. The innovative interventions included in this initiative have the potential to improve the health outcomes of youth and young adults who are living with HIV.

The ETAC at UCLA aims to complete the analysis and dissemination of findings, best practices, and lessons learned from using social media and mobile technology to support the engagement of HIV-positive youth and young adults in medical care by the end of 2019. The ETAC hopes that the findings will serve to inform future policy and practices for programs seeking to use ever-changing and improving social media platforms and mobile technology in the delivery of high quality, culturally appropriate HIV primary health care interventions. Successful scale-up of these types of interventions will require understanding how and why youth and young adults use social media and emerging mobile technologies for personal health.

## Appendix

Multimedia Appendix 1 Demonstration site interventions.

## Abbreviations

ACASI: audio computer-assisted self-interview
ETAC: Evaluation and Technical Assistance Center
HHS: Health and Human Services
HIPAA: Health Insurance Portability and Accountability Act
HRSA: Health Resources and Services Administration
MSM: men who have sex with men
RWHAP: Ryan White HIV/AIDS Program
SMI: Using Social Media to Improve Engagement, Retention, and Health Outcomes along the HIV Care Continuum initiative
SMS: short message services
SPNS: Special Projects of National Significance
UCLA: University of California, Los Angeles
