# Authorship Correction: Promising Approaches for Engaging Youth and Young Adults Living with HIV in HIV Primary Care Using Social Media and Mobile Technology Interventions: Protocol for the SPNS Social Media Initiative

**DOI:** 10.2196/15660

**Published:** 2019-09-09

**Authors:** Melissa Medich, Dallas T Swendeman, W Scott Comulada, Uyen H Kao, Janet J Myers, Ronald A Brooks, Special Projects of National Significance Social Media Initiative Study Group

**Affiliations:** 1 Department of Family Medicine University of California, Los Angeles Los Angeles, CA United States; 2 Department of Psychiatry and Biobehavioral Sciences University of California, Los Angeles Los Angeles, CA United States; 3 Department of Medicine University of California San Francisco San Francisco, CA United States

The authors of “Promising Approaches for Engaging Youth and Young Adults Living with HIV in HIV Primary Care Using Social Media and Mobile Technology Interventions: Protocol for the SPNS Social Media Initiative” (JMIR Res Protoc 2019;8(1):e10681) have added the Special Projects of National Significance Social Media Initiative Study Group as a group author on the original publication in last position. The new list of authors is as follows:

Melissa Medich, Dallas T Swendeman, W Scott Comulada, Uyen H Kao, Janet J Myers, Ronald A Brooks, Special Projects of National Significance Social Media Initiative Study Group

The corrections will appear in the online version of the paper on the JMIR website on September 9, 2019, together with the publication of this correction notice. Because this was made after submission to PubMed, PubMed Central, and other full-text repositories, the corrected article, including collaborators associated with the group author, also has been resubmitted to those repositories.

