# Supplementary material for: Promising Approaches for Engaging Youth and Young Adults Living with HIV in HIV Primary Care Using Social Media and Mobile Technology Interventions: Protocol for the SPNS Social Media Initiative
Source: JMIR Res Protoc. 2019 Jan 31;8(1):e10681. doi: 10.2196/10681 (PMC6374729; doi:10.2196/10681)
Supplement: Multimedia Appendix 1 [file resprot_v8i1e10681_app1.pdf]

**Multimedia Appendix 1.** Demonstration site interventions.

| Demonstration Site | Name of Intervention | Brief Narrative |
|---|---|---|
| Coastal Bend Wellness Foundation | **WELLNESS WEB 2.0** | Text-messaging service delivering adapted ARTAS (antiretroviral treatment access study) messages and/or Mobile Wellness messages focused on engagement and retention in HIV care, skills building, utilization of support services |
| Friends Research Institute, Inc. | **"Text Me, Girl!"** | Unidirectional, 270 pre-written, theory-based, trans-specific text messages (i.e., three per day) targeted, tailored, and personalized for HIV-positive young trans women. |
| Health Research, Inc. (NYAI) New York State Department of Health AIDS Institute | **Y Get It?** www.ygetit.org | Web-based mobile application with various components. A patient-to-patient social networking platform for anonymous chats moderated by a peer engagement and educator professionals ("PEEPs"); reminders, email and SMS services; Instagram Serial focused on young characters and their life stories. |
| Howard Brown Health Center | **SMARTEE** (Social Media App for Retention, Treatment, Engagement, and Education) | A modified Healthvana mobile application with text messaging, access to electronic health records, appointment and medication reminders, and access to support services. |
| MetroHealth System | **Positive Peers Application** (PPA) www.positivepeers.org | Web-based, mobile application featuring self-management tools and social networking including a private Facebook group, anonymous users and avatars, a gaming component, and links to supportive resources. |
| Penn State University | **OPT-In For Life** https://optinforlife.org | Web-based, HIPAA compliant, mobile application providing secure communications with medical case managers, medication reminders, test results, and a list of clinic locations, and supportive services. |
| Philadelphia FIGHT | **Positively Connected for Health** http://iknowushould2.com | App based intervention with several components: a digital health literacy workshop, called **TreatYourSelf;** a web-based media campaign encouraging testing and linkage to care, **iknowUshould2;** a mobile application, **Positively SmART**, for text messaging; and a digital health literacy program component, **APPlify Your Health**. |
| Public Health Foundation Enterprises (SFDPH) San Francisco Department of Public Health | **Health eNav** www.healthenavigation.org | Web-based and digital navigation services that provides user-generated content, bi-directional text messaging with a digital case manager, medication and appointment reminders, electronic medication adherence monitors, and referrals for supportive services. An integrated dashboard allows digital navigators to geo-locate, identify, and communicate with patients. |
| Wake Forest University | **WeCare** | Bi-directional, live, text messaging, FB/FB messenger and GPS-based social networking mobile application that provides appointment and medicine reminders, and problem solving for issues related to engagement in HIV care. |
| The Washington University St. Louis | **e-Volution** | Text messaging with medical case managers, the Epharmix mobile application providing medication reminders, access to electronic medical lab results, and a virtual, anonymous online support group. |
